# Optical Etching to Pattern Microstructures on Plastics by Vacuum Ultraviolet Light

**DOI:** 10.3390/ma13092206

**Published:** 2020-05-11

**Authors:** Tomotaka Doi, Takatoki Yamamoto

**Affiliations:** Department of Mechanical Engineering, Tokyo Institute of Technology, Meguro-ku, Tokyo 152-8550, Japan; t.d.19.march@gmail.com

**Keywords:** vacuum ultraviolet light, etching, microfabrication, nanofabrication, patterning, room temperature and pressure

## Abstract

We proposed and demonstrated an optical dry etching method for transferring a pattern on a photomask to a surface of plastics by decomposing the irradiated area using the high energy of vacuum ultraviolet light (VUV) at room temperature and pressure. Two kinds of wavelengths of 160 nm and 172 nm were used as the vacuum ultraviolet light, and the patterning performances for polymethyl methacrylate (PMMA) and polycarbonate (PC) were compared. As a result, it was revealed that proportional relationships were obtained between the etching rate and the irradiation dose for both wavelengths, and the cross-sectional profiles were anisotropic. In addition, both PMMA and PC were etched at a wavelength of 160 nm, whereas PC could not be etched at a wavelength of 172 nm, suggesting that it correlates with the bond dissociation energies of the molecular bonds of the materials and the energies of the photons. Furthermore, by combining this method with the optical bonding method that we had previously developed to bond surfaces irradiated with VUV, we have demonstrated a method for fabricating microfluidic devices by irradiating only with VUV. This paper shows that this technique is a new microfabrication method suitable for simple and mass production of plastic materials.

## 1. Introduction

Microfluidics deals with the precise control and manipulation of fluids constrained to a typically sub-millimeter scale and takes advantage of scaling law applied to various physical effects. They have been the subject of active research in several fields, such as biotechnology, medical care, and energy [1,2,3,4,5]. The applications of microfluidic devices have constantly expanded since the birth of the concept in the 1970s, not only owing to development-based needs but also due to advancements made in processing technology, such as the application of refined processes and new materials [6,7].

Low cost disposable devices required for medical applications and wearable flexible devices have come to be widely used. These include plastics, which have a low unit price despite their excellent mechanical and chemical properties. The major fabrication methods for microfluidic devices using these plastics are classified into the following: (1) removal processes such as cutting and laser ablation [7,8,9,10]; (2) injection molding [11,12]; (3) replica molding [13,14,15]; (4) additive manufacturing typified by the 3D printer [16,17]; and (4) embossing processes including nanoimprinting [18,19,20,21]. However, general cutting and additive manufacturing techniques fall short of the processing accuracy required by several tens of microns as well as difficulties encountered in achieving consistent mass production. On the other hand, injection molding and embossing are excellent in terms of mass production and depending on the mold, nano-scale structures can be fabricated just like the nanoimprint method. Replica molding is inferior to injection molding in terms of mass production, whereas it has an advantage for prototyping. However, the manufacturing of high-precision molds is extremely difficult and expensive.

Therefore, in this study, we have proposed and experimentally demonstrated an optical etching method using vacuum ultraviolet light (VUV) as a microfabrication method for plastics; this is a process capable of delivering simple and consistent mass production. The optical etching method described here is categorized as a dry-etching method, the processing takes place without any vacuum condition which is usually required for conventional dry etching. A photomask is irradiated with VUV, and the pattern on the photomask is transferred to the surface of the plastic by decomposing only the irradiated part using high energy light at a room temperature and pressure. Furthermore, by combining this with the optical bonding method we previously developed to bond surfaces by irradiating VUV onto the bonding surfaces [22,23,24], we have also demonstrated a new method for fabricating microdevices by only irradiating with VUV.

## 2. Materials and Methods

**Figure 1:**
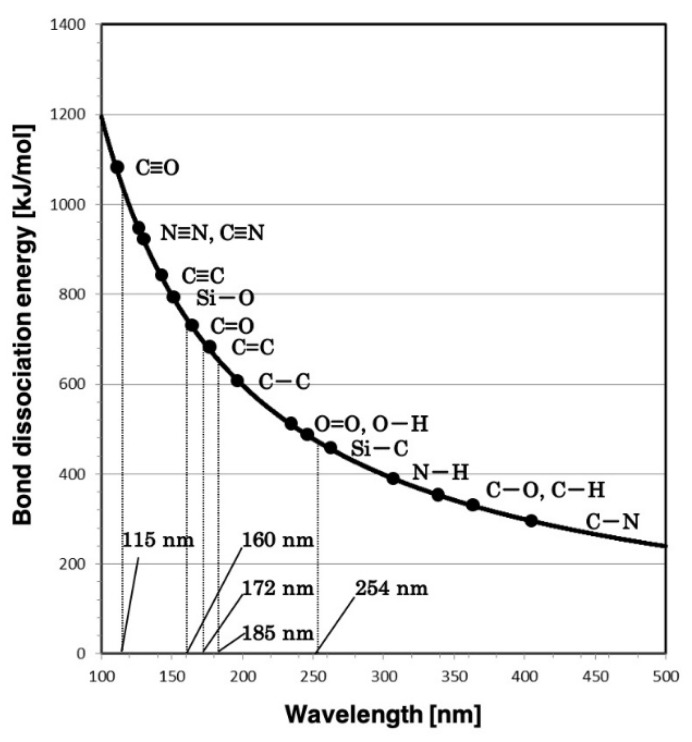
Relationship between bond dissociation energy of organic molecular bonds and wavelength of photon (photon energy).

The energy of light is inversely proportional to its wavelength. VUV has a very short wavelength of between 100 nm and 200 nm, and therefore has high energy (approximately 600 to 1200 kJ/mol). Figure 1 shows the relationship between photon energy and wavelength [25,26]. The two types of light sources used in this study had peak wavelengths of 160 nm and 172 nm. The bond dissociation energy of typical molecular bonds that constitute plastic are shown, and it can be seen that single bonds such as C–C, C–O, and C–H have a lower bond dissociation energy than the photon energy associated with VUV wavelengths of 172 nm and 160 nm. The bond dissociation energy of the double bond C=O falls within the energy range associated with wavelengths of 172 nm and 160 nm. Such molecular bonds on the surface of the material can be broken by irradiating with VUV with energy higher than the bond dissociation energy [27,28,29,30].

On the other hand, it is known that when the atmosphere is irradiated with VUV, oxygen molecules absorb the light energy and generate various reactive oxygen species, which can cause the molecular bonds to be broken due to oxidation by these reactive oxygen species under atmospheric conditions [23,24]. These physical effects are already being used for optical cleaning of organic stains on material surfaces [31], an optical bonding method that directly bonds plastic surfaces that are excited by VUV irradiation [32,33], and microstructure fabrication using the vitrification of silicone by irradiation with VUV [22]. In the present study, these decomposing effects of organic materials are used for etching on the plastic surface.

Two types of light sources for the VUV were used: a xenon excimer lamp with a wavelength of 172 nm (L12530-01, Hamamatsu Photonics, Hamamatsu, Japan), and a deuterium lamp with a peak wavelength of 160 nm (L12462, Hamamatsu Photonics, Hamamatsu, Japan). Although the light spectrum of the deuterium lamp ranges from 120 nm to 170 nm, since about 60% of the light output is concentrated within the wavelength of 160 ± 10 nm, it was used as a light source with a wavelength of 160 nm in this study.

A thin film (100 nm thick) of chromium patterned on synthetic quartz (1 mm thick) with high transmittance in the VUV region was used as the photomask. The amount of exposure dose was calculated from the transmittance of synthetic quartz, which is 0.85 at a wavelength of 172 nm and 0.20 at a wavelength of 160 nm, and the measured data from the exposure meter (UIT-150, USHIO INC., Tokyo, Japan). Two kinds of materials, polymethyl methacrylate (PMMA) and polycarbonate (PC) were used as etching materials, both of which have excellent light transmittance and are often used as microfluidic devices. PMMA has C=O bonds with binding dissociation energy higher than 172 nm light energy and lower than 160 nm light energy. PC also has a C=O bond, but the molecular weight of PC is higher than that of PMMA, so that the molecular weight dependency in etching can be evaluated. Therefore, PMMA and PC should be good tools for investigating the relationship between the energy of light and the bond dissociation energy.

**Figure 2:**
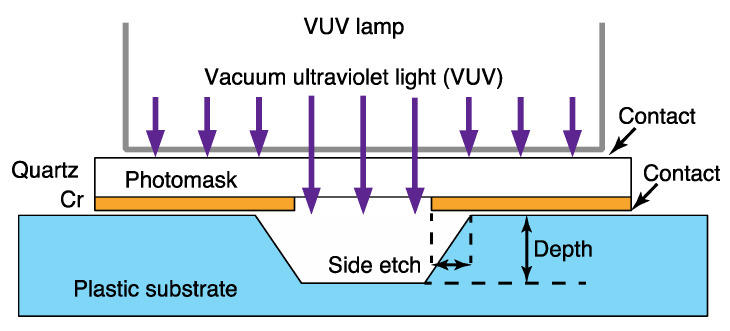
Schematic of the experimental method.

The experimental method is shown in Figure 2. We used two types of masking. One is a method using a photomask, and the other is a method for making a mask layer directly on the substrate surface. The substrate was irradiated with VUV through the photomask with the light source, the substrate and photomask in close contact in the case of using photomask. Even though the surfaces of the substrates were flat, a small gap of a few µm remained between the photomask and substrates, and it is presumed that oxygen in this gap turns into reactive oxygen species, which contributes to the etching process. On the other hand, the directly deposited mask was used, in order to investigate the effect of the small gap on patterning.

The shapes of the surface of the substrate before and after processing were measured with an atomic force microscope (VN-8000, KEYENCE, Osaka, Japan), and the cross-sectional shapes after etching were evaluated to determine the etching rate of the optical etching.

## 3. Results

### 3.1. Optical Etching

**Figure 3:**
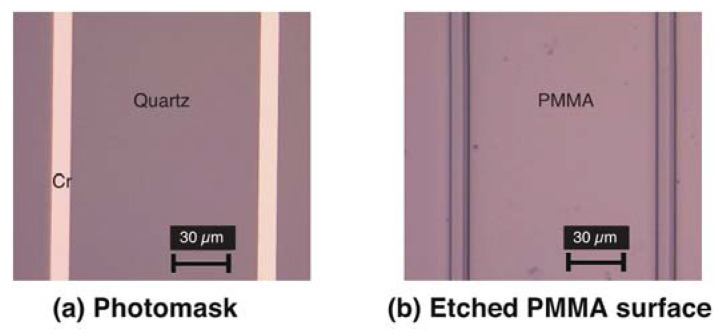
Typical result of optical etching, (**a**) magnified photograph of the photomask, (**b**) etched surface on polymethyl methacrylate (PMMA).

We investigated various patterns for the photomask sizing from several hundred µm to 500 nm. Figure 3a shows the typical design used as the photomask with line and space patterns of 10 µm and 100 µm respectively. Figure 3b is a magnified photograph of the surface of the PMMA substrate after being exposed to 800 mJ of VUV with a wavelength of 160 nm. The patterns from the photomask can be clearly seen to have been transferred to the PMMA substrate by VUV irradiation.

**Figure 4:**
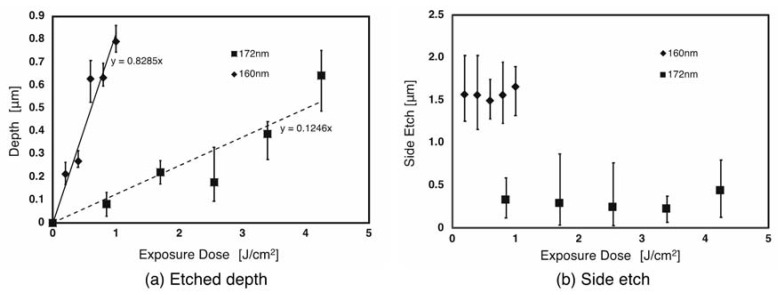
Results of optical etching of PMMA, (**a**) etched depth and (**b**) side etching.

**Figure 5:**
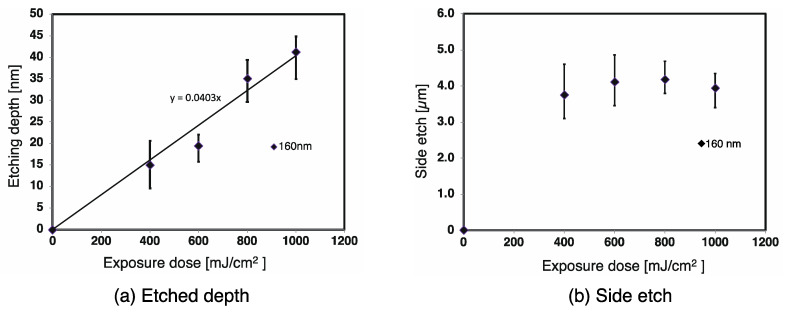
Results of optical etching of PC, (**a**) etched depth and (**b**) side etching.

The atomic force microscope measurements of the etching depth and the side etching length are shown in Figure 4. The exposure dose and etching depth are seen to have a linear relationship for both PMMA and PC. Figure 4 shows that the average etching rate for PMMA was 0.12 µm/J at a wavelength of 172 nm and 0.83 µm/J at a wavelength of 160 nm (Figure 4a). The side etching length however, despite some variation, was found to be fairly constant regardless of the exposure dose, being about 0.5 to 1.0 µm at a wavelength of 172 nm and about 1.2 to 1.8 µm at a wavelength of 160 nm (Figure 4b). Figure 5 shows the same measurements for PC at a wavelength of 160 nm only, as etching could not be confirmed at a wavelength of 172 nm. The etching rate was 0.03 nm/J, which is lower than that of PMMA as shown in Figure 5a. The side etching length was again fairly constant regardless of the exposure dose at between 3 and 4.5 µm as shown in Figure 5b.

### 3.2. Fabrication of Microfluidic Device by Optical Etching and Bonding

**Figure 6:**
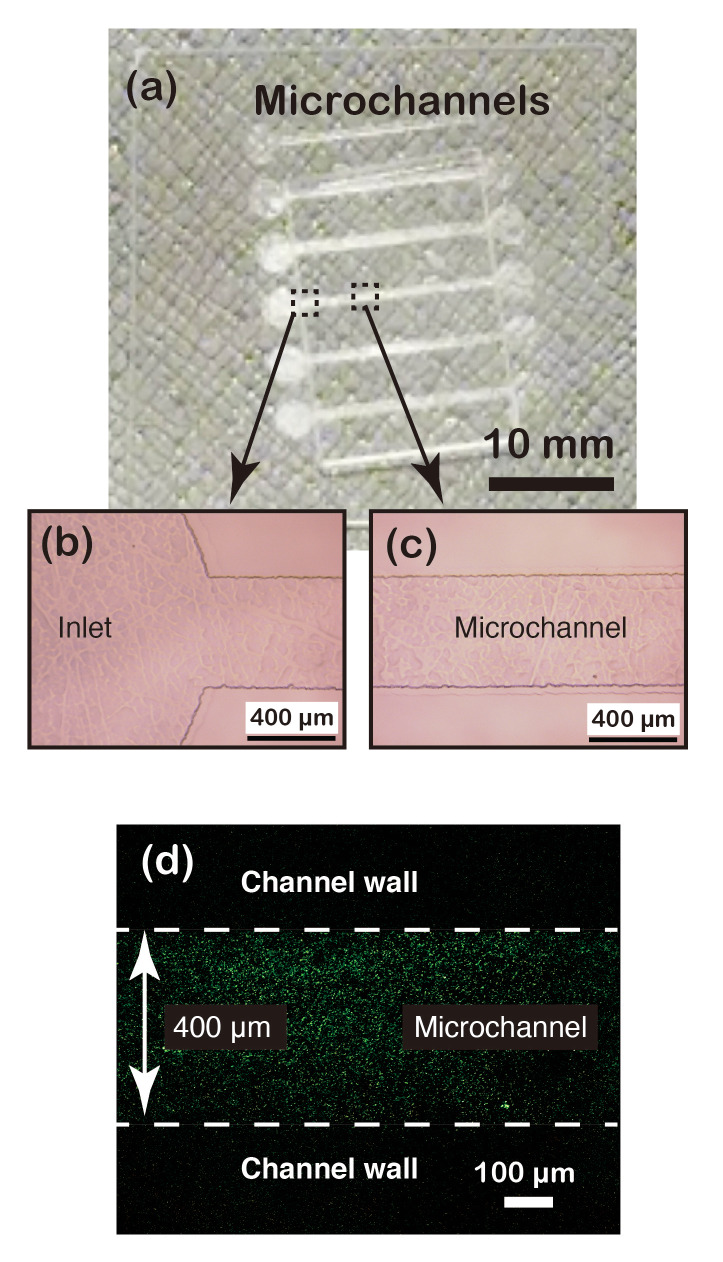
Fabrication of microfluidic device by optical etching and bonding: (**a**) fabricated microchannels, (**b**) magnified view of the inlet part, (**c**) magnified view of channel part, and (**d**) fluorescent particles flowing inside the microchannel.

We made a prototype of a PMMA made microfluidic device using only VUV irradiation. The results of the present study show that plastic etching is possible with VUV, and our previous research shows that plastic surfaces can be directly bonded by mere exposure to VUV (optical bonding method) [20,21]. We therefore tried to demonstrate that plastic-made microfluidic devices can be fabricated with VUV, through a combination of optical bonding and optical etching methods. The PMMA surface was exposed to 3 J/cm^2^ of VUV at a wavelength of 160 nm through a photomask with a channel pattern. Figure 6a–c show the resulting channel structures with a depth of about 3 µm fabricated on the substrate. VUV irradiation at 60 mJ/cm^2^ and a wavelength of 160 nm on the bonding surface of the channel substrate, and on the surface of another PMMA substrate, enabled the two substrates to be bonded together to create a closed microchannel device. A pressure of 5.0 MPa was applied during bonding to ensure contact between the substrates.

The bonding efficacy of the fabricated device was evaluated by feeding a solution into the microchannel, containing fluorescent particles (S1392, Invitrogen) at the concentration of 10^11^ particle/mL, the results of which are shown in Figure 6d. The figure shows that the fluorescent particles are only present in the channel portion, confirming that the surfaces are sealed uniformly.

## 4. Discussion

The etching depth of PMMA was deeper at a wavelength of 160 nm than at 172 nm. When organic materials were irradiated with VUV at a wavelength of 172 nm, a reduction in methyl groups was observed [19,24]. Similarly, when organic materials were irradiated with VUV at a wavelength of 160 nm, not only was there a reduction in methyl groups but also a decrease in C=O bonds [21], which is greater than the energy of VUV at a wavelength of 172 nm but lower than the energy at 160 nm. Moreover, the absorptivity of PMMA at 160 nm was found to be more than double that at 172 nm [21]. It is not quite clear why the etching rate of PC should be lower than that of PMMA; however, the difference in etching rates for the two wavelengths may be attributed to these effects. 

**Figure 7:**
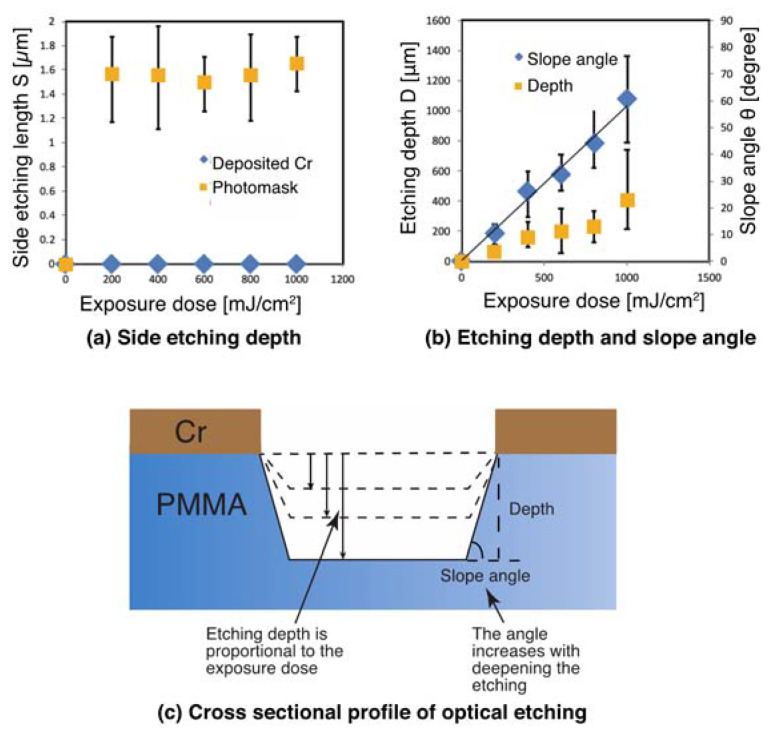
Optical etching (**a**) side etching difference between photomask and deposited Cr, (**b**) etching rate versus slope angle, (**c**) schematic of the etched cross-sectional profile.

The side etching length was fairly constant in both PMMA and PC, irrespective of the etching depth. The reason that the side etch length was greater than the etching depth could be due to poor contact between the photomask and substrate because of the unevenness of the substrate surface. To confirm this, optical etching was conducted by directly depositing and patterning a Cr layer on the PMMA substrate by vacuum deposition using a stencil mask. This meant that the optical etching was done by ensuring no gaps between the photomask and the substrate surface during exposure. The results in Figure 7a and b show that there are no discernible side etchings detected. The side etching seen in Figure 4 and Figure 5 is believed to be caused by light intrusion due to poor contact between the photomask and substrate. As shown in Figure 7b, the slope angle increases with exposure dose and resulting etching depth, indicating that the etching proceeds as shown in Figure 7c. These results suggest that deep etching with a high aspect ratio may be possible depending on the exposure dose.

## 5. Conclusions

In this study, a VUV at two different wavelengths was used to demonstrate an optical etching method that is suitable for mass production on account of its simple process and large area of exposure. In the case of VUV at a wavelength of 160 nm, the etching was successful on both PMMA and PC, although there were differences in etching rates. It was also found that the etching depth is proportional to the exposure dose and that the etching is anisotropic. Etching by irradiation at a wavelength of 172 nm was possible on PMMA but not on PC. It was deduced that the optical etching success dependence on wavelength is due to factors such as the bond dissociation energy of the molecular bonds that make up the material, and the photon energy (directly related to the wavelength), so further work will be done to study these relationships in more detail. Future studies will also include research into the role of photon energy and reactive oxygen species in the etching process. 

Furthermore, by combining the optical etching method proposed in this study with the optical bonding method established in our previous research, we succeeded in developing a new method to fabricate devices using only VUV irradiation through the example of fabricating a microfluidic device.

In addition to organic structural materials, this method will be used to develop the patterning of functional organic materials such as organic electronic materials in the future, as well as developing a new microfabrication method for organic microdevices using light.
